# Network properties of salmonella epidemics

**DOI:** 10.1038/s41598-019-42582-3

**Published:** 2019-04-16

**Authors:** Oliver M. Cliff, Vitali Sintchenko, Tania C. Sorrell, Kiranmayi Vadlamudi, Natalia McLean, Mikhail Prokopenko

**Affiliations:** 1 Centre for Complex Systems, Faculty of Engineering and IT, University of Sydney, Sydney, NSW 2006 Australia; 2 Centre for Infectious Diseases and Microbiology-Public Health, Institute of Clinical Pathology and Medical Research, NSW Health Pathology, Westmead Hospital, Sydney, NSW 2145 Australia; 3 University of Sydney Marie Bashir Institute for Infectious Diseases and Biosecurity, University of Sydney, NSW 2006 and Westmead Institute for Medical Research, Sydney, NSW 2145 Australia

## Abstract

We examine non-typhoidal Salmonella (S. Typhimurium or STM) epidemics as complex systems, driven by evolution and interactions of diverse microbial strains, and focus on emergence of successful strains. Our findings challenge the established view that seasonal epidemics are associated with random sets of co-circulating STM genotypes. We use high-resolution molecular genotyping data comprising 17,107 STM isolates representing nine consecutive seasonal epidemics in Australia, genotyped by multiple-locus variable-number tandem-repeats analysis (MLVA). From these data, we infer weighted undirected networks based on distances between the MLVA profiles, depicting epidemics as networks of individual bacterial strains. The network analysis demonstrated dichotomy in STM populations which split into two distinct genetic branches, with markedly different prevalences. This distinction revealed the emergence of dominant STM strains defined by their local network topological properties, such as centrality, while correlating the development of new epidemics with global network features, such as small-world propensity.

## Introduction

Non-typhoidal *Salmonella* causes an estimated 93.8 million human cases of salmonellosis and over 155,000 deaths globally each year^1–3^. *Salmonella enterica* subsp. *enterica* serovar Typhimurium (*S*. Typhimurium or STM) has been the dominant cause of non-typhoidal human salmonellosis worldwide^3,4^. It is evolving continuously, persisting and undergoing adaptation within different ecological niches. STM has demonstrated remarkable diversity as a zoonotic ‘generalist’ serovar of public health importance, from which epidemics and ‘specialist’ high virulence strains emerge^5–7^. Whilst the impact of changes in STM diversity on disease incidence has been recognised^4,7–9^, the drivers of STM population dynamics during seasonal epidemics remain poorly understood^4^. Advances in high-resolution genotyping have highlighted limitations in traditional phylogenetic approaches to the analysis of non-hierarchical relationships between recombining strains within species, which could not be represented as bifurcating trees^10,11^. In this study, we examine STM epidemics as complex systems characterised by non-linear interactions of diverse microbial strains, and describe the process of emergence of successful strains. Our results challenge the established view that STM epidemics are caused by random sets of co-circulating STM genotypes preferentially occurring during the summer months^12,13^ and demonstrate that network properties of evolving STM strains can correlate with the development of new epidemics in unexpected ways.

Recent accumulation of representative sets of molecular subtyping data has provided an opportunity to examine the intricate connectivity of co-circulating STM strains. We used a collection of 17,107 STM isolates identified in the New South Wales (NSW) State Salmonella Reference Laboratory in Sydney, Australia between 1 January 2008 and 31 December 2016. This set contained 99.3% of all STM isolated from human cases throughout NSW during this period. All isolates were genotyped by multiple-locus variable-number tandem-repeats analysis (MLVA). A “tandem-repeat” is defined as a pattern of several nucleotides which is repeated and the repetitions are directly adjacent to each other. The MLVA profile is defined as a string of integers representing the numbers of repeats in several fixed genetic locations (loci), e.g., 3-9-7-12-523. Thus, MLVA profiles consist of the total numbers of tandem repeats in each of five loci. Crucially, the differences between bacterial strains captured in MLVA profiles have proven sufficiently discriminatory for public health laboratory surveillance and outbreak investigations^14,15^. There are 1675 unique MLVA profiles obtained over 3,287 days in this dataset.

By interrogating genotypes identified during nine consecutive seasonal epidemics, we have been able to quantify heterogeneity, interconnectedness and temporal frequency of STM isolates associated with unique or common MLVA profiles. In particular, we observed a heavy-tail distribution in the prevalence of MLVA profiles (see Fig. S2 in Supplementary Information). Table S1 (Supplementary Information) details the commonest MLVA profiles, ordered by decreasing prevalence.

Empirical networks were constructed where each MLVA profile was represented as a node in a graph, and the edge weight between nodes was defined as the Manhattan distance between profiles. Using these networks, we then examined the global network topology and evolution and its relationship to the emergence of dominant or successful STM strains^16,17^. In addition to the global network, a different network was created for every date in the dataset. That is, for a given date, a separate network for all unique MLVA profiles was built within a moving window of 365 days (Supplementary Information), capturing annual periodicity and seasonal patterns of the consecutive epidemics. For each such network, the clustering, path length, and small world coefficient were computed^18,19^, yielding a moving average of these network characteristics.

**Figure 1:**
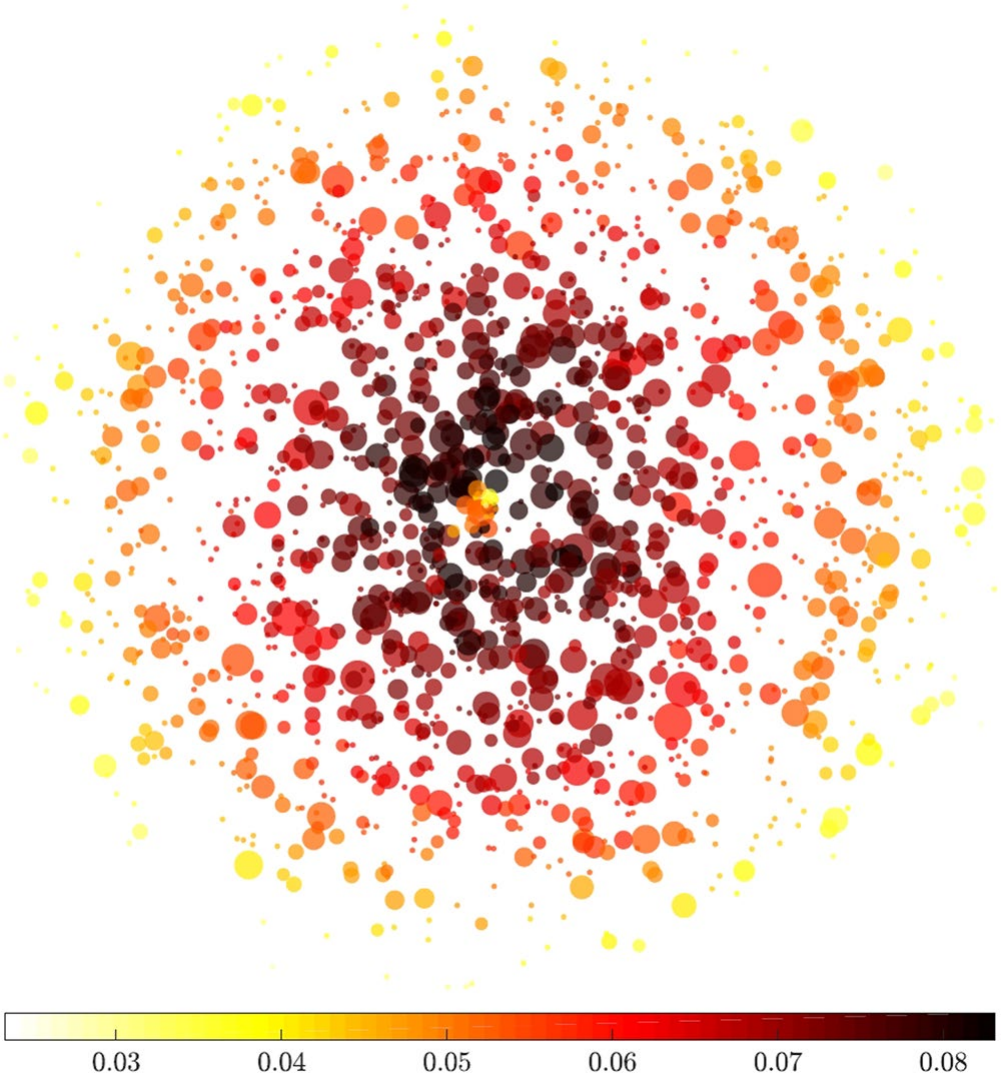
The STM MLVA network, where the edge weight between nodes is represented by the L_1_-norm distance between them. The size of each node is set in proportion to the prevalence of the corresponding MLVA profile. The network layout is given by a simple spring algorithm; moreover, the edges in the graph are removed for readability and each node is coloured by its closeness centrality.

**Figure 2:**
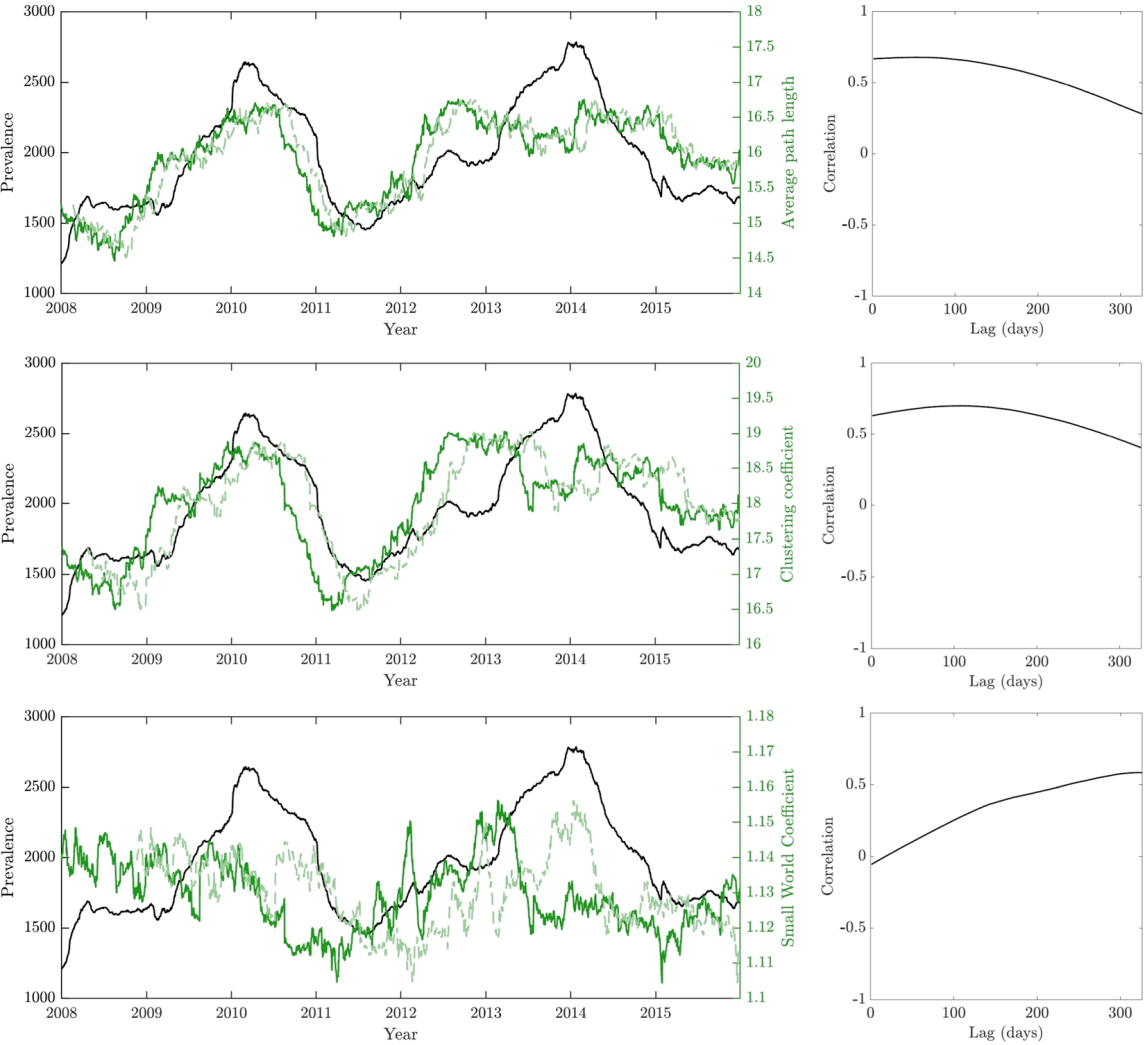
The relationships between networks characteristics and *S*. Typhimurium prevalence. The yearly prevalence of cases is represented as black lines, i.e. the number of cases within 365 days of the date on the x-axis (a new network is computed based on all instances within 365 days of the date on the x-axis, in order to capture annual periodicity and seasonal yearly patterns of the consecutive epidemics). For each row, relation of different MLVA network characteristics (for that year) to the yearly prevalence is shown. The characteristic path length (top row, A), the average clustering coefficient (middle row, B), and the small world coefficient (bottom row, C) are plotted in solid green. The right-hand figures illustrate the sample correlation coefficients between each network measure and the yearly prevalence for lags of the time series (of up to 365 days, within the time series over 3,287 days). The time lag that produces the maximum correlation for each measure is recorded, and the corresponding lagged time series is then overlaid in light green, in order to compare with the actual time series (shown in black).

The topology of individual networks reflected evolving characteristics of STM populations. Figure 1 demonstrates the connectivity and complexity of such networks, highlighting the high variability of individual closeness centrality values across the individual MLVA profiles (nodes). The clustering coefficient and small world coefficient of each node also vary across the entire set of characterised MLVA types, as shown in Fig. S3. The corresponding average network properties, such as path length, centrality and small-world coefficients, characterize different dimensions of the diversity within STM populations, and correlated well with the prevalence of STM infections over time, with respect to their prevalence measured as a moving average with annual periodicity (Fig. 2). Specifically, correlations peak at ~300 days for the small world coefficient (and much earlier for the average clustering coefficient at ~50 days and the characteristic path length at ~100 days) (Fig. 2). This suggests that the STM activity might be heralded well in advance by small changes in the network topology.

Network nodes, i.e. MLVA profiles, were then clustered into groups within which the nodes were more similar to each other than to those in other groups. To examine the similarity of MLVA profiles, we employed overlapping and partitioning clustering methods (Fig. S1, see Supplementary Information). The overlapping approach clustered all nodes within a certain threshold distance of a focus node as part of the same cluster thus allowing a node to participate in more than one cluster. Table S2 presents the commonest MLVA profiles, ordered by decreasing average prevalence of the overlapping cluster to which the MLVA belongs. The partitioning (or mutually exclusive) approach allows for any case of STM disease with a specific MLVA profile to be part of only one cluster.

**Figure 3:**
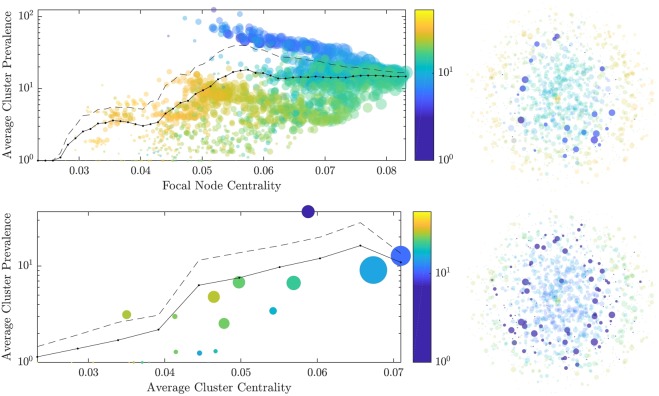
The closeness centrality of MLVA profiles is compared to the prevalence of their clusters. The clusters are obtained by either the overlapping (top row) or the partitioning (bottom row) algorithms. For overlapping clusters (threshold distance five), the *individual* centrality of each node is plotted against the logarithm of average prevalence of their cluster. For partitioning clustering (with 21 mutually exclusive clusters), the *average* centrality of each cluster is plotted against the logarithm of average prevalence of that cluster. The size of each circle (left subfigures) is set in proportion to the size of the corresponding cluster. Solid lines trace binned averages, using: 50 equal size bins in the range between 0.0233 and 0.0831 for the top sub-figure (i.e., individual node centrality); and 10 equal size bins in the range between 0.0233 and 0.0710 for the bottom sub-figure (i.e., average cluster centrality). Dashed lines trace the corresponding standard deviations above the means. The scattergrams are coloured by the pairwise L_1_-norm between nodes (top left) or clusters (bottom left). That is, for the overlapping approach (top left), each node is coloured according to distance to other focal MLVAs, whereas in the partitioning approach (bottom left), the average pairwise distance between all nodes in two clusters is used. The left subfigures show how far these clusters are, in terms of these distances, from the most prevalent cluster, with a clear colour gradient. The right subfigures visualise the nodes within the most prevalent cluster as opaque in each network, with all other nodes semi-transparent. The size of each node (right subfigures) is set in proportion to the prevalence of the corresponding MLVA profile.

These clusters were used to evaluate the relationship between the position of STM genotypes within the network and their potential to cause outbreaks. Specifically, the closeness centrality of MLVA profiles was compared with the prevalence of their clusters (Fig. 3). The network centrality measures represented the relative impact of isolates with different MLVA profiles in the epidemic, and allowed us to trace the evolutionary drift of strains towards more prevalent cases in terms of their centrality. The graph clustering algorithms identify potential clusters for every MLVA profile. In Fig. 3, for the overlapping approach we set the threshold distance as five, accounting for at least a detectable mutation in all loci before the MLVA profiles are considered distinct. The threshold distance to define a mutually exclusive cluster was chosen to maintain concordance between the average sizes of clusters identified by two different approaches, rather than similarity in the number of clusters. As a result, 21 mutually exclusive clusters were identified, and the diversity of the STM population and the relative abundance of clustered isolates were quantified; the majority represented community-acquired outbreaks with or without an epidemiologically-confirmed source.

Crucially, we observed a non-linear relationship between the centrality of nodes and their prevalence, i.e., their success as a food-borne human pathogen. The most prevalent clusters were of medium centrality, where branching occurred at the highest centrality nodes. The shift in direction of association between centrality and cluster prevalence appeared for clusters with an average prevalence of 10 (Fig. 3, left). This shift suggested a significant change in STM virulence or transmissibility. The gradient of colouring indicated an evolution from the high centrality nodes towards this upper branch. There were two distinct genetic branches, one of lower prevalence/severity, and one of higher. There was the transition from sporadic STM strains represented by MLVA profiles with a low cluster density and node centrality into highly ‘successful’ strains causing outbreaks and represented by MLVA profiles with high cluster density and medium node centrality. The most ‘successful’ STM strains seemed to emerge from MLVA profiles with the highest centrality in the network, via a reduction of their centrality, towards less central but more prevalent profiles.

**Figure 4:**
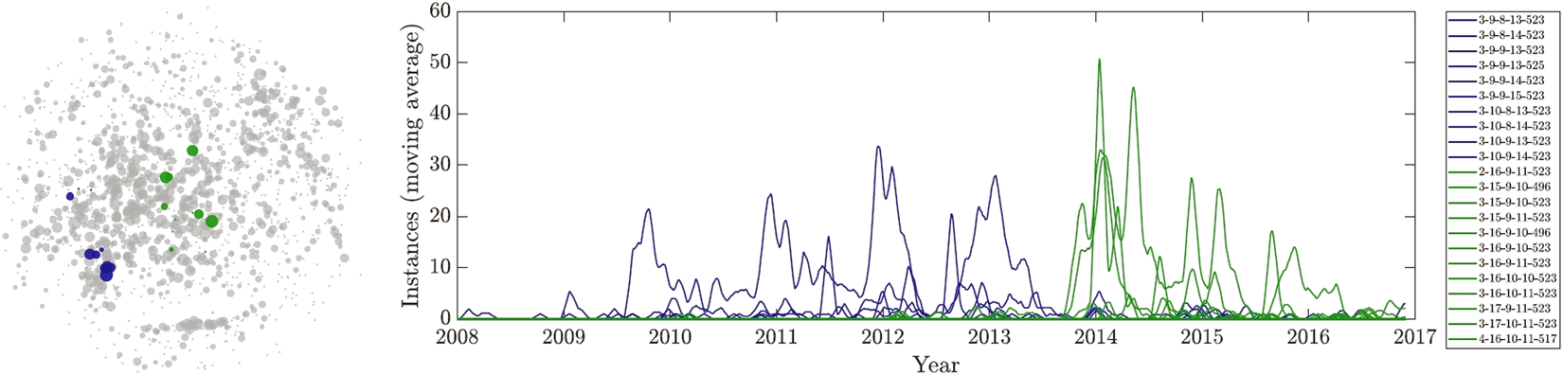
Temporal evolution of two STM MLVA clusters, which are successful in occupying a niche at different times (the clusters were obtained through the partitioning algorithm with 450 resultant clusters). The left subfigure shows the nodes in the two clusters. The size of each node is set in proportion to the prevalence of the corresponding MLVA profile. The right subfigure shows the corresponding individual time series, in terms of the average cluster prevalence (i.e., the moving average of instances of the identified STM isolates). Strains in the blue cluster show seasonal patterns beginning in 2009, but become markedly less prominent in 2013; the cluster is characterised by 9 to 10 tandem-repeats in the second locus, and the corresponding MLVA profiles exhibit a medium network centrality. Strains in the green cluster strongly emerge at the end of 2013, and reduce in prominence at the end of 2017; the cluster is distinguished by 15 to 17 tandem-repeats in the second locus, and its MLVA profiles show a high network centrality.

To investigate the temporal evolution of epidemics and the role of individual strains, the entropy of the MLVA frequency distribution within 30-day time intervals was assessed. It appeared that the STM population diversity was gradually increasing and oscillating, suggesting the expansion to different niches in the process of nine seasonal epidemics (Fig. S4). MLVA clusters also evolved over time with the replacement of one successful STM strain by another at the end of the epidemic. This is shown in Fig. 4 where the partitioning algorithm was used to create 450 mutually exclusive clusters. This analysis revealed a major shift in the population of epidemic strains in 2014 with the replacement of previously endemic STM strains by new ones occupying different positions in the network. These findings are concordant with the observation of gradual replacement of STM phage type 135 with STM phage types 170 and 9 in New South Wales over the study period^9^. Figure S5 illustrates the time series of different MLVA clusters over time. Time series were obtained by taking a (30 day) moving average of the number of instances of each MLVA profile (i.e., their prevalence), and suggested that different clusters were more prevalent at different times, possibly due to variations in their prevalence in relevant zoonotic reservoirs, and herd immunity in human hosts.

In this study, we inferred undirected STM networks from surveillance and molecular genotyping data representing nine consecutive seasonal epidemics of salmonellosis in Australia, quantified the diversity and variability of these evolving genetic networks, correlated their small-world network properties with the severity of STM epidemics in Australia; and identified distinct evolutionary branches in terms of the network nodes’ centrality. These findings enhance and broaden our view of epidemics of salmonellosis and support the feasibility and added value of network analysis of relationships between diverse bacterial strains within the same species. This approach is aligned to the niche theory as it treats the impact of individual variants (STM MLVA profiles in this case) on the population as proportional to their frequency in the population^20^. Our results also provide a new platform for public health surveillance. In contrast to existing mechanistic approaches based on the search for pathogens with matching genotypes, it highlights the added value of monitoring of ongoing STM population diversity and the identification of new genotypes as reservoirs from which future epidemics might emerge.

With increasing evidence of diversification in pathogen genomes in response to evolutionary pressure and human interventions^6,7,13^ it is essential to improve the quality and resolution of public health surveillance. The network analysis targets microbial genotypes as operational units of biological and surveillance function. The increasing uptake of whole genome sequencing for public health surveillance and availability of microbial genome data in public repositories strengthen the utility of network analyses. The emergence of successful STM strains leading to a summer epidemic can be signified as a reduction in newly identified MLVA types in the preceding winter and spring^9^. Representation of epidemics as networks of individual strains adapting in order to maximize their chances of propagation in a hostile environment offers an alternative and powerful approach to monitor the dynamics of seasonal epidemic. It reveals fundamental architectural features of pathogen networks and ascertains empirical indicators of the proximity to tipping points in bacterial populations^21,22^. Even small changes over time in small-world coefficients, path length and clustering of the networks can be instructive for the prediction of the temporal increases in disease prevalence. They quantify the fitness of invading populations and pave the way for a more systematic assessment of the structural and dynamic properties of epidemics and anticipation of critical transitions in disease incidence^23–26^, providing early warning signs through disease surveillance and thus enabling improvements in emergency preparedness and response^27,28^.

## Supplementary information

Supplementary Information

Supplementary Information - Video

## Data Availability

The dataset describes the entire collection of 17,107 STM isolates identified in the New South Wales (NSW) State Salmonella Reference Laboratory in Sydney, Australia between 1 January 2008 and 31 December 2016. This dataset contains data on several outbreaks which are still under investigation with legal proceedings pending which involve food producers and groups of patients, and so it will become available once these proceedings are finalized.
