# Supplementary material for: Network properties of salmonella epidemics
Source: Sci Rep. 2019 Apr 16;9:6159. doi: 10.1038/s41598-019-42582-3 (PMC6467889; doi:10.1038/s41598-019-42582-3)
Supplement: Supplementary file 1 — Supplementary Information [file 41598_2019_42582_MOESM1_ESM.pdf]

Supplementary Information for

Network Properties of Salmonella Epidemics

Oliver M. Cliff, Vitali Sintchenko, Tania C. Sorrell, Kiranmayi Vadlamudi, Natalia McLean,

Mikhail Prokopenko

Supplementary text: data and methods

Figures S1 – S5

Table S1 – S2

Movie S1

Supplementary text: data and methods

The dataset for this paper comprised instances of *S*. Typhimurium in New South Wales, Australia. The methods we used are common to network theory and will be outlined below. Most of the analysis was done using MATLAB (MathWorks) inbuilt functions.

We construct one global network, using all data, as well as multiple daily networks each corresponding to a specific day of the dataset.

For each daily network, we compute the clustering, path length, and small worldness – this effectively yields a *moving average* of these network characteristics. The measures are then compared to a (yearly) moving average of the prevalence (computed by counting the number of cases within the same time window for every day of the dataset). The graph layout (i.e., the spring algorithm) was provided by Cytoscape, an open source platform for network analysis.

Dataset: MLVA of *S.* Typhimurium in NSW, Australia

All STM isolates referred to the NSW Enteric Reference Laboratory at the Centre for Infectious Diseases and Microbiology, NSW Health Pathology in Sydney, Australia, for typing between 1st of January 2008 and 31 December 2016 were included in the study and genotyped using MLVA.

The multiplex Polymerase Chain Reaction (PCR) was performed to amplify the variable number tandem repeats (STTR9, STTR5, STTR6, STTR10pl and STTR3) and subsequent analyses were conducted as described previously[1]. MLVA results were reported as a string of five numbers representing relevant repeats[2].

The *S*. Typhimurium reference strain LT2 (GenBank NC_003197) was used as a control throughout and consistently generated the expected MLVA type 4-13-13-10-0211. A total of 17,107 human STM isolates were examined following removal of duplicate isolates from the same episode of the disease (99.3% of all STM cases recorded in NSW during the study period).

Video S1 illustrates the dataset, by highlighting the STM cases recorded in 2008 in NSW, including Sydney, Australia.

The MLVA profile is given by the vector $\boldsymbol{p} = \{p_1, p_2, \ldots, p_5\}$ where the subscript denotes the loci, e.g., p_1 is the number of repeats in locus 1.

Network and Clustering

The network topology is a complete graph where we represent each MLVA profile as a node, and the edge weight between nodes is given by the Manhattan distance between profiles.

Clustering methods. The two clustering methods used are: overlapping and partitioning, as shown in Figure S1.

Edge Weights. For this work, we require edge weights (or, equivalently, a dissimilarity measure) between all pairs of MLVA profiles. The proper choice of an edge weight w_{ij} between a pair of nodes i and j depends on the properties of the system.

Let $\boldsymbol{p}_i$ and $\boldsymbol{p}_j$ be the the MLVA profiles for nodes i and j. Then, the L_1-norm distance d_{ij}, or the Manhattan distance, between these nodes is computed as

$$d_{ij} = \left|\boldsymbol{p}_i - \boldsymbol{p}_j\right|_1 = \left|\boldsymbol{p}_{i1} - \boldsymbol{p}_{j1}\right| + \left|\boldsymbol{p}_{i2} - \boldsymbol{p}_{j2}\right| + \cdots + \left|\boldsymbol{p}_{i5} - \boldsymbol{p}_{j5}\right|.$$

An alternative similarity measure is given by the L_2-norm distance:

$$d_{ij} = \left|\boldsymbol{p}_i - \boldsymbol{p}_j\right|_2 = \sqrt{\left(\boldsymbol{p}_{i1} - \boldsymbol{p}_{j1}\right)^2 + \left(\boldsymbol{p}_{i2} - \boldsymbol{p}_{j2}\right)^2 + \ldots + \left(\boldsymbol{p}_{i5} - \boldsymbol{p}_{j5}\right)^2}.$$

The L_1-norm is selected as the similarity measure between two MLVA $\boldsymbol{p}_i$ and $\boldsymbol{p}_j$ in order to represent the expectation that a single large difference in one individual locus of $\boldsymbol{p}_i$ and $\boldsymbol{p}_j$

represents a smaller overall distance than a combination of several small differences between individual loci of $\boldsymbol{p}_i$ and $\boldsymbol{p}_j$. For example, MLVA pattern {3-12-12-9-523} is expected to be more similar to {3-12-12-11-523} than to {3-13-13-10-523}. In this example, the L_1-norm for the first pair results is 2, i.e., the sum of differences in the 4th locus, being *smaller* than the L_1-norm for the second pair which yields 3 across three different inner loci. On the other hand, the L_2-norm would also produce distance 2 for the first pair (i.e., a square root of the squared difference in the 4th locus), but would yield distance $\sqrt{3}$ for the second pair, i.e. a square root of the sum of three individual minimal distances across three inner loci, which is *greater* than 2, contradicting our expectation.

In our analysis, we paid a special attention to locus 5. Rather than treating it as any other vector element of $\boldsymbol{p}_i$, we follow a normalization procedure which combines the approach of Larsson et al.[3] (that is, explicitly identifying and summing the corresponding numbers of tandem repeats in 27 and 33 base-pairs, when these numbers are available) with a division of $\boldsymbol{p}_{i5}$ by 37 and rounding the result, when these numbers are not available. The edge weights are given by the reciprocal of these distances, i.e.,

$$w_{ij} = 1/d_{ij}.$$

Computing the weights in this way results in them being normalised. The edge weights are used to compute a number of network measures; moreover, they are employed by Cytoscape to lay out the network via a force-directed spring algorithm.

Overlapping and Partitioning Clustering. Using the edge weights, we obtained the overlapping clustering algorithm simply by including all nodes within a certain distance. For instance, let C_i be the cluster associated with node *i*, then

$$C_i = \{j: d_{ij} \leq D_{max}\}$$

for some threshold D_{max}. In this work we set $D_{max} = 5$ such that we account for a detectable mutation in all loci.

We used hierarchical agglomerative clustering, which begins with all nodes in separate clusters and greedily combines them according to a dissimilarity criterion. The dissimilarity requires a "metric" and a "linkage criteria". The metric gives the distance between pairwise observations (i.e., the L_1-norm), and the linkage criterion d_{IJ} determines the distance between two *sets* of observations *I* and *J*. We employed the Ward's minimum variance method in this study[4].

An example. It is instructive to consider MLVA profile 3-9-7-12-622 which appears as the most frequent profile in terms of the average cluster prevalence (overlapping clusters), with the average cluster prevalence of 124.7714286 (see Table S2). The individual prevalence of this profile is only 1 (that is, it has been identified only once in 8 years), and so it is important to trace the reasons behind its high average cluster prevalence. The overlapping cluster, within the threshold distance D_{max} = 5, to which the profile 3-9-7-12-622 belongs includes 35 MLVA profiles overall. In order to determine its cluster neighbours, we firstly consider the normalisation of the profile 3-9-7-12-622 – since it does not appear in the study of Larsson et al.[3], the corresponding numbers of tandem repeats in 27 and 33 base pairs are not available, and hence, the value $\boldsymbol{p}_{i5}$ is obtained by division of 622 by 37 and rounding the result, yielding 3-9-7-12-17. This normalised profile is, therefore, within D_{max} = 5 to several profiles with very high individual frequency: specifically, the top 4 most frequent profiles are in the same overlapping clusters (see Table S1). In these profiles the value $\boldsymbol{p}_{i5}$ = 523 is replaced by 14 = 2 + 12, as there are 2 base-pairs 27 and 12 base-pairs 33, according to Larsson et al.[3] Thus, the average cluster prevalence of the profile 3-9-7-12-622 (after normalisation: 3-9-7-12-17) is high because it belongs to an overlapping cluster which also includes profiles with high individual prevalence, while these profiles themselves belong to overlapping clusters with smaller average cluster prevalence.

Network Measures

Clustering and Closeness. In capturing characteristics of the weighted networks inferred in this study, we utilize several measures: the clustering coefficient and closeness centrality (both local and global), as well as the characteristic path length (a global measure). We employ the weighted versions of these measures, following recent studies of brain networks[5]. We chose closeness centrality, rather than betweenness centrality[6], percolation centrality[7], or PageRank centrality[8], due to a natural connection between the normalized closeness centrality and the characteristic path length, with the latter being the average of the former, as shown below. Since the networks are fully connected, the unweighted degree distribution of each node is equivalent, reducing the immediate applicability of other measures, such as the network assortativity[9,10] and the rich-club coefficient[11], which need to be carefully adapted for weighted networks[12].

The clustering coefficient is typically defined for unweighted networks to measure the degree to which a nodes' neighbours are connected. There have been numerous proposals for

applying this measure to weighted networks, all designed to capture different network characteristics. We employ the approach of Onnela et al. [13]:

$$c_i = \frac{1}{k_i\,(k_i - 1)} \sum_{j,k} (\hat{w}_{i,j}\hat{w}_{j,k}\hat{w}_{i,k})^{1/3}$$

where $\hat{w}_{i,j} = w_{i,j} / \max(w)$, and k_i is the number of edges connected to n_i. A common global measure of clustering in a network is given by averaging the local coefficients,

$$C = \frac{1}{N} \sum_i c_i$$

where N is the number of nodes in the network. This is known as the average clustering coefficient.

The closeness centrality of a node is computed as the sum of the length of the shortest paths between nodes. In this work we use the normalized form of this measure:

$$l_i = \frac{N}{\sum_j d_{i,j}}$$

The characteristic path length is a robust measure for quantifying the average distance between nodes. It is computed as follows:

$$L = \frac{1}{N(N-1)} \sum_{i \neq j} d_{i,j}$$

which is equivalent to taking the average closeness centrality.

We can combine the above measures by taking the ratio of the clustering coefficient to the closeness centrality c_i / l_i - similarly, the global form is given as C/L. This ratio relates to another common measure in network theory, known as the *small world coefficient*[14]. The small world coefficient is often used to capture the degree to which a network can be characterised as having the "small-world" phenomena[15]. Typically for the small world coefficient, however, C and L are taken relative to the random network clustering coefficient C_{rand} and path length L_{rand}. That is,

$$\sigma = \frac{C/C_{rand}}{L/L_{rand}}$$

Given the MLVA network is complete, there is no random structure; however, random edge weights for a complete network would result in $C_{rand} \approx 1$ and $L_{rand} \approx 1$. Thus, the ratio C/L could be considered a proxy to σ. Various other centrality measures, including PageRank and betweenness centrality, yielded similar results for tracing the evolution of strains, as well as predicting the severity of a given MLVA profile. However, we only presented results for the clustering and closeness of a node due to their intuitive description and our focus in small-world properties.

Entropy

Given the frequency p_i of each MLVA profile i, observed during a specific time interval T, we obtain an MLVA frequency distribution. Then the entropy H_T of this MLVA frequency distribution is measured as

$$H_T = -\sum_i p_i \log p_i$$

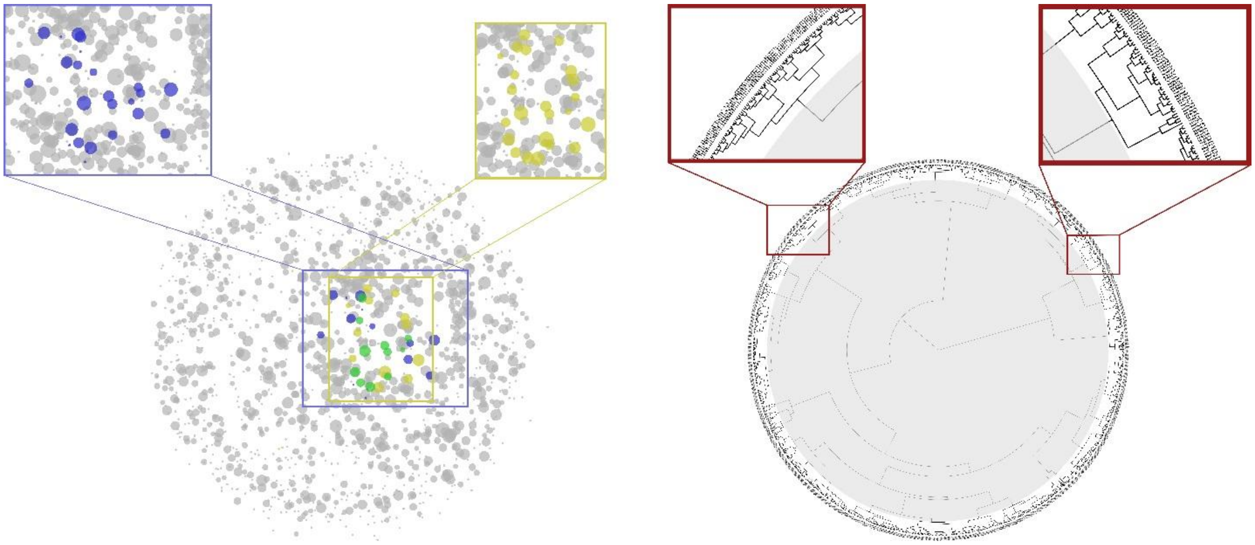

Figure S1. Clustering the MLVA profiles. The two clustering methods used are: overlapping (left) and partitioning (right). The size of each node is set in proportion to the prevalence of the corresponding MLVA profile. The overlapping approach (left) is to cluster all nodes within a certain distance to a focus node, within the same cluster. We show two clusters (a blue and a yellow cluster), where the green nodes correspond to both. The partitioning approach (right) uses agglomerative clustering to yield non-overlapping clusters. We use a distance threshold, and if the nodes are further away than this threshold on the dendrogram, they become separate clusters. The figure shows the polar dendrogram of the entire MLVA dataset, with insets showing two separate clusters, and grey shading denoting the distance threshold.

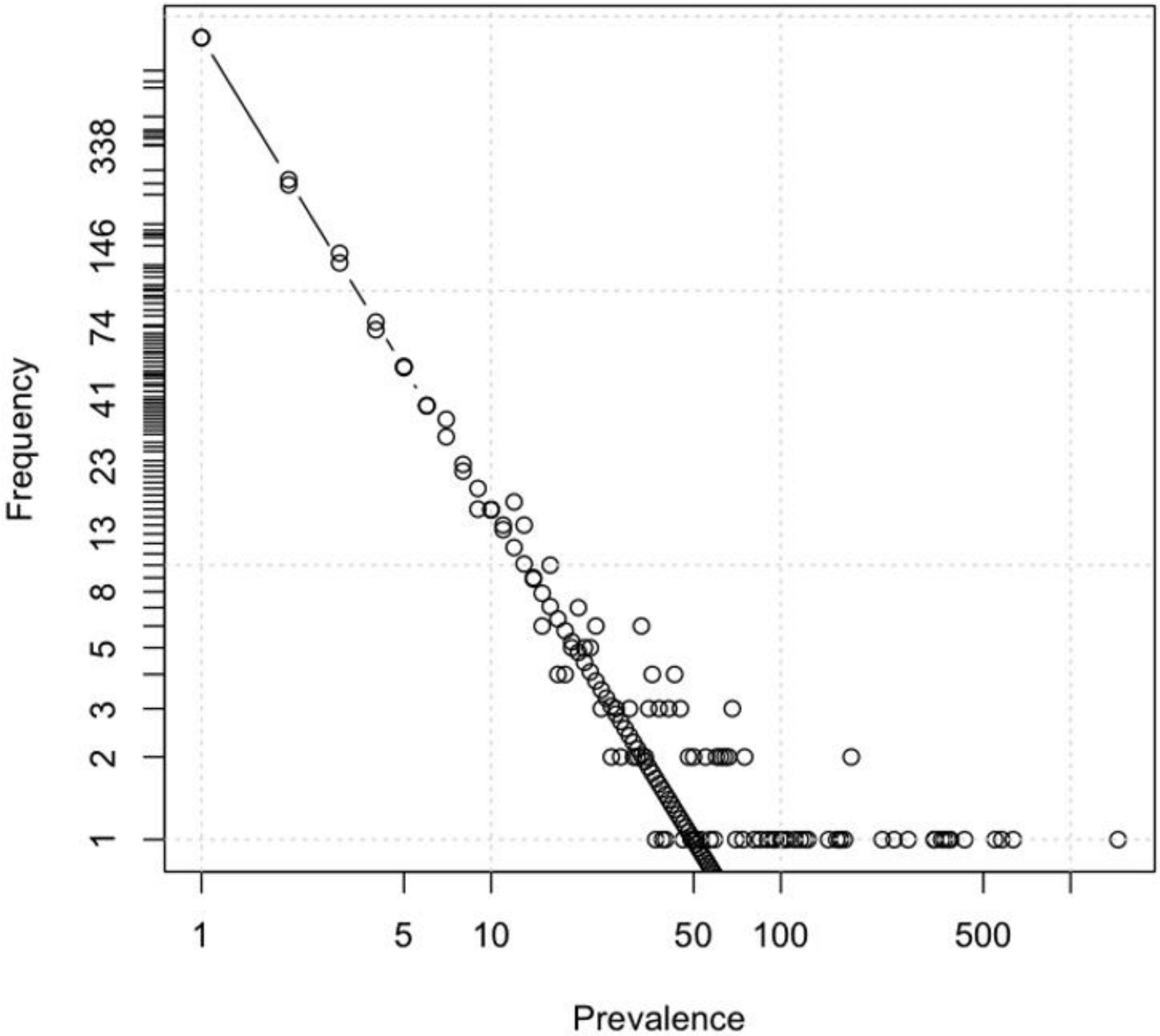

Figure S2. Frequency vs prevalence of MLVAs on a log-log scale, showing a heavy tail common in high-variance distributions, including power laws. The exponent of a power law fit is estimated as *s* = -1.72238, with standard deviation of 0.0161 (t value of -106.9; probability $Pr(>|t|) < 2e\text{-}16$).

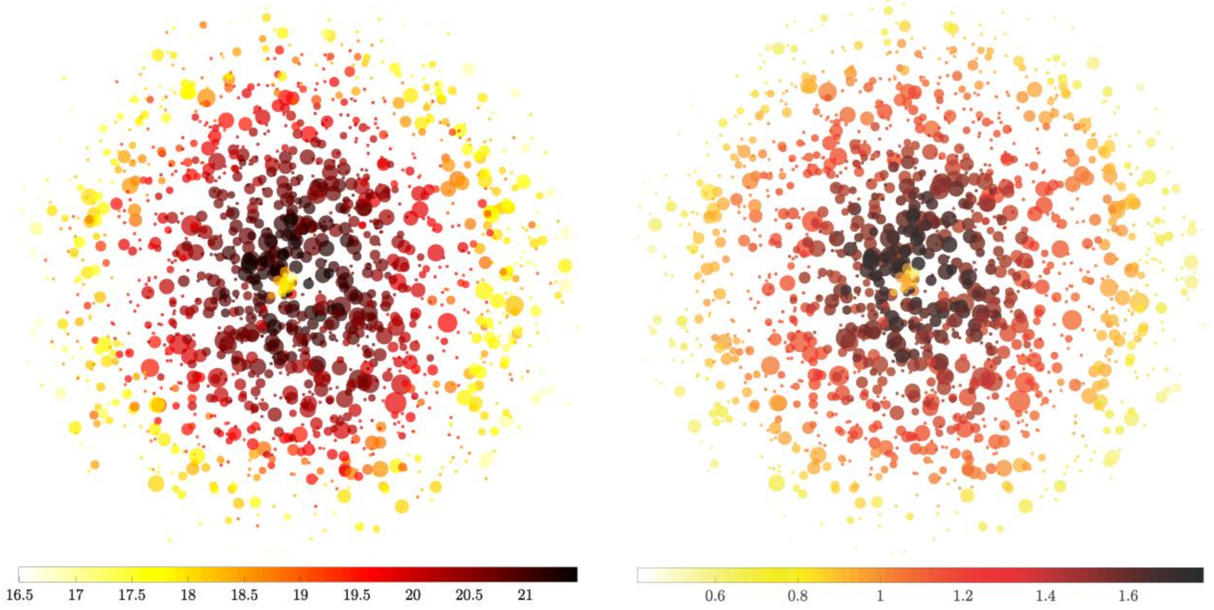

Figure S3. The MLVA network, where the edge weight between nodes is given by the L_1-norm distance between them. The size of each node is set in proportion to the prevalence of the corresponding MLVA profile. The network layout is given by a simple spring algorithm; moreover, the edges in the graph are removed for readability and each node is coloured by its attribute: clustering coefficient (left), and small world coefficient (right).

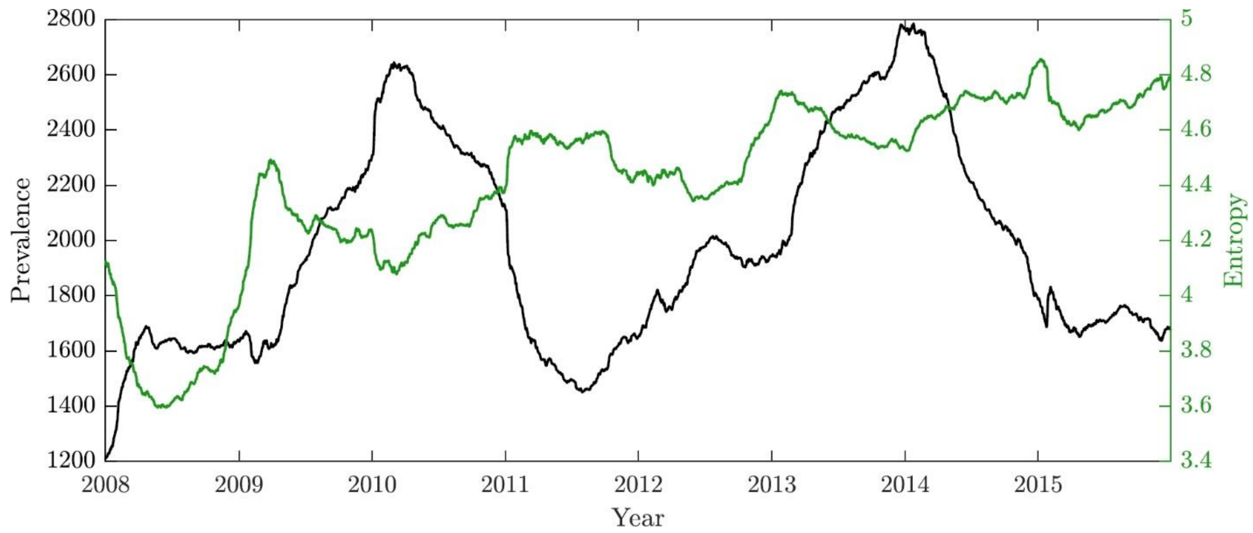

Figure S4. MLVA profile entropy versus S. Typhimurium prevalence. The black curve traces the prevalence. The entropy of the MLVA frequency distribution, measured in bits, shown in green, is computed using the moving average approach, over 30 days (within the time series over 3,287 days). The diversity of S. Typhimurium is increasing and oscillating, suggesting that more of the search space is being explored.

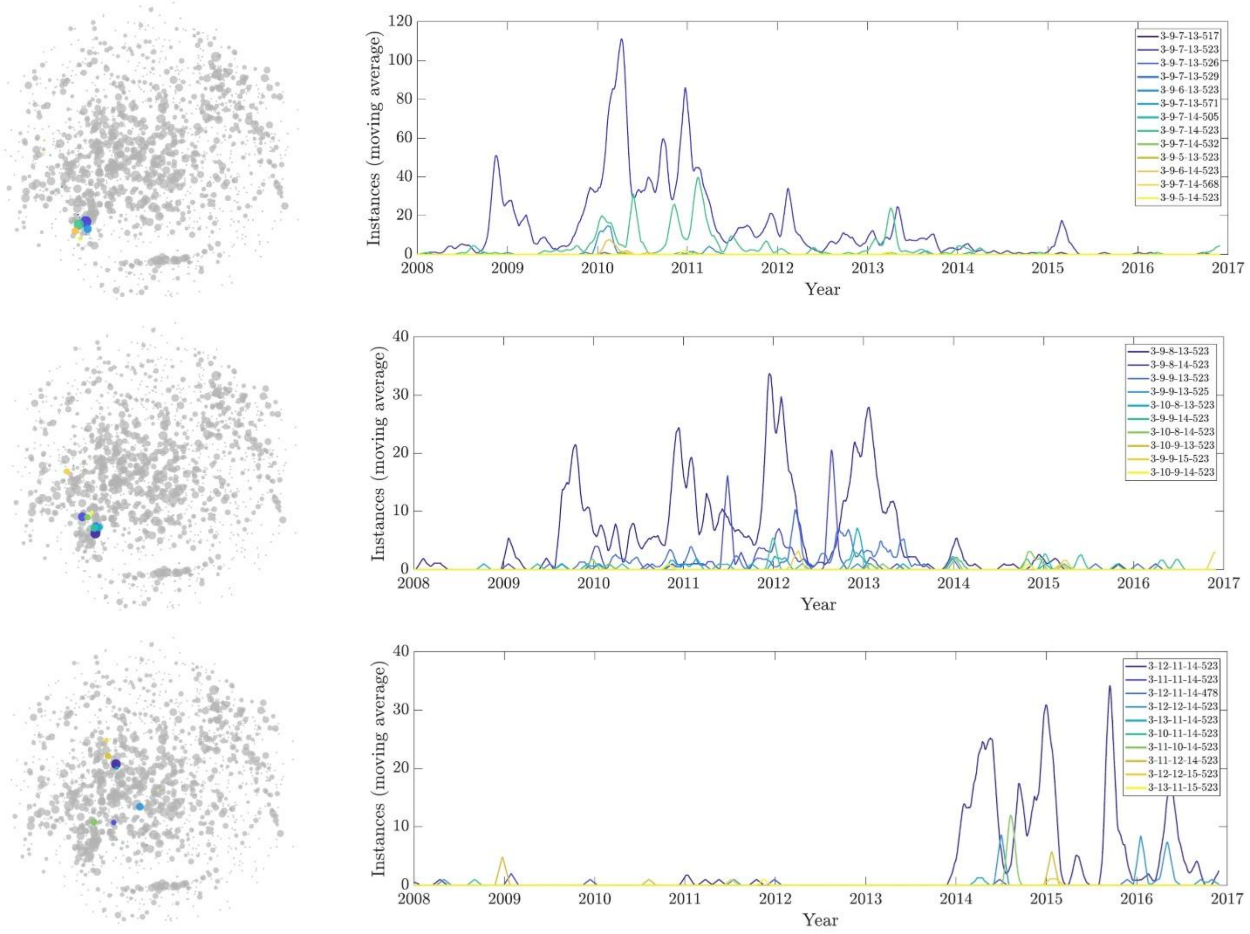

Figure S5. Temporal evolution of MLVA clusters, obtained through the partitioning algorithm (with 450 resultant clusters). Each row shows the time series of a unique cluster, demonstrating the different niches being acquired in time by different strains, in terms of their prevalence (i.e., the moving average of instances of the identified STM isolates). The left subfigures show the nodes in each cluster. The size of each node is set in proportion to the prevalence of the corresponding MLVA profile. The right subfigures show the individual time series. The MLVA profiles are coloured according to their L_1-norm to the most prevalent node within their cluster.

Table S1. Most frequent MLVA profiles, ordered by decreasing individual prevalence, alongside their centrality in the constructed STM network, and the first and last year of MLVA identification.

MLVA profile	Prevalence	Average cluster prevalence	Centrality	First year	Last year
3-9-7-13-523	1459	43.109375	0.059602884	2008	2016
3-9-8-12-523	634	39.64150943	0.064200176	2008	2016
3-9-7-12-523	579	43.07913669	0.061406719	2008	2016
3-9-8-13-523	549	42.13103448	0.062240356	2008	2015
3-17-9-11-523	431	17.07518797	0.075568566	2012	2016
3-10-8-9-523	385	33.74137931	0.066400222	2008	2015
3-17-9-12-523	379	17.34057971	0.074577778	2012	2016
3-9-7-14-523	368	45.6173913	0.057191547	2008	2016
3-12-12-9-523	359	13.70621469	0.078013855	2008	2016
3-12-11-14-523	341	13.90714286	0.072877307	2008	2016
3-12-9-10-550	338	18.08843537	0.071776884	2008	2013
3-26-13-8-523	275	15.8	0.049270341	2014	2016
3-10-14-12-496	246	13.33027523	0.068874933	2008	2015
3-10-14-11-496	224	11.82608696	0.069724923	2008	2016
3-16-9-11-523	175	16.44	0.078024737	2010	2016
3-9-7-15-523	175	56.12087912	0.054560234	2009	2013
3-24-13-10-523	166	9.852941176	0.056622237	2013	2016
3-13-11-9-523	161	14.6043956	0.079900957	2010	2016
3-16-9-12-523	160	16.36931818	0.076976008	2012	2015
3-10-7-12-523	156	40.67096774	0.064605552	2008	2016
3-12-15-13-523	146	13.2892562	0.07109567	2010	2015
3-17-10-11-523	124	16.10596026	0.077728368	2013	2016
3-9-9-12-523	121	38.46706587	0.066587302	2008	2016
4-15-11-0-490	117	7.703389831	0.053269841	2008	2016
3-10-7-13-523	112	42.2	0.062621287	2008	2015
3-10-8-12-523	105	35.2962963	0.067699508	2008	2016
3-9-9-13-523	102	39.39597315	0.064476465	2009	2016
3-12-13-9-523	100	11.47058824	0.076923077	2010	2016
3-14-11-12-523	96	15.59545455	0.082452951	2010	2012
3-10-15-12-496	94	13.46296296	0.066545051	2009	2013

Table S2. Most frequent MLVA profiles, ordered by the decreasing average cluster prevalence, alongside their centrality in the constructed STM network.

MLVA profile	Average cluster prevalence	Prevalence	Cluster size	Centrality
3-9-7-12-622	124.7714286	1	35	0.05209724
3-9-8-12-424	109.0810811	1	37	0.056346541
3-9-7-18-523	95.66666667	1	24	0.047336944
3-9-4-12-523	90.55102041	35	49	0.053019053
3-9-7-17-523	85.13953488	5	43	0.049573104
3-10-4-13-523	77.40816327	1	49	0.053918576
3-7-8-14-523	74.375	3	64	0.053465031
5-9-8-14-523	71.92424242	2	66	0.056318174
5-9-7-12-523	71.3	1	70	0.057921988
3-9-7-16-523	70.47761194	11	67	0.051982652
5-9-7-14-523	69.42647059	2	68	0.05416048
3-9-7-13-571	69.38356164	1	73	0.053509359
3-8-7-15-523	68.47826087	5	69	0.051873377
3-9-8-16-523	67.98571429	1	70	0.053967131
3-7-7-13-523	66.71052632	8	76	0.053468438
5-9-7-13-523	66.69736842	2	76	0.056321955
3-9-7-14-430	66.50704225	1	71	0.0535418
5-9-8-13-523	66.36	3	75	0.058659023
3-9-5-14-523	66.22222222	2	72	0.052153913
3-9-5-13-523	65.55128205	1	78	0.054155236
4-9-6-13-523	64.74683544	1	79	0.055760476
3-9-8-12-463	63.55844156	1	77	0.0596283
3-9-8-13-430	63.11392405	1	79	0.057929987
3-9-8-13-457	63.11392405	1	79	0.057929987
3-10-6-13-496	62.05063291	1	79	0.058462825
3-9-7-8-523	59.84375	2	64	0.058487278
3-8-10-14-523	59.30555556	1	72	0.059528878
3-8-7-14-523	58.6091954	1	87	0.05425329
3-8-7-11-523	58.08247423	2	97	0.058622135
3-9-7-14-568	57.31460674	1	89	0.054337619

Movie S1. STM cases recorded in 2008 in NSW, including Sydney, Australia.

References.

1. Doolittle, W. F. Phylogenetic classification and the universal tree. *Science* 284, 2124–2129 (1999).
2. Gemeinholzer, B. Phylogenetic networks. In: *Analysis of biological networks*. Junker, B. H. & Schreiber, F., Eds (Wiley Series in Bioinformatics, Wiley InterScience, New Jersey) 255–281 (2008).
3. Larsson, J. T. et al. Development of a new nomenclature for Salmonella Typhimurium multilocus variable number of tandem repeats analysis (MLVA). *Eurosurveillance* **14,** pii:19174 (2009).
4. Ward, J. H. Jr. Hierarchical Grouping to Optimize an Objective Function, *J. Am. Stat. Assoc.* **58**, 236–244 (1963).
5. Muldoon, S. F., Bridgeford, E. W. & Bassett, D. S. Small-World Propensity and Weighted Brain Networks. *Sci. Rep.* 6, 22057 (2016).
6. Freeman, L. C. A set of measures of centrality based on betweenness. *Sociometry* 40, 35–41 (1977).
7. Piraveenan, M., Prokopenko, M. & Hossain, L. Percolation centrality: Quantifying graph-theoretic impact of nodes during percolation in networks. *PLOS ONE* **8(1)**, e53095 (2013).
8. Page, L., Brin, S., Motwani, R. & Winograd, T. The PageRank citation ranking: bringing order to the web. Technical Report, Stanford InfoLab (1999).
9. Piraveenan, M., Prokopenko, M. & Zomaya, A. Y. Assortativeness and information in scale-free networks. *Eur. Phys. J. B* **67**, 291–300 (2009).
10. Piraveenan, M., Prokopenko, M. & Zomaya, A. Y. Assortative mixing in directed biological networks. *IEEE/ACM Trans. Comput. Biol. Bioinform.* **9(1)**, 66–78 (2012).
11. Zhou, S. & Mondragón, R. J. The rich-club phenomenon in the Internet topology. *IEEE Commun. Lett.* **8**, 180–182 (2004).
12. Alstott, J., Panzarasa, P., Rubinov, M., Bullmore, E. T. & Vértes, P. E. A unifying framework for measuring weighted rich clubs. *Sci. Rep.* **4**, 7258 (2014).
13. Onnela, J.-P., Saramäki, J., Kertész, J. & Kaski, K. Intensity and coherence of motifs in weighted complex networks. *Phys. Rev. E* 71, 065103 (2005).
14. Telesford, Q. K., Joyce, K. E., Hayasaka, S., Burdette, J. H. & Laurienti, P. J. The Ubiquity of Small-World Networks. *Brain Connect.* 1, 367 (2011).
15. Watts, D. J. & Strogatz, S. H. Collective dynamics of 'small-world' networks. *Nature* 393, 440 (1998).
